# Creep Behavior of High-Strength Concrete Subjected to Elevated Temperatures

**DOI:** 10.3390/ma10070781

**Published:** 2017-07-11

**Authors:** Minho Yoon, Gyuyong Kim, Youngsun Kim, Taegyu Lee, Gyeongcheol Choe, Euichul Hwang, Jeongsoo Nam

**Affiliations:** 1 Department of Architectural Engineering, Chungnam National University, 99 Daehak-ro, Yuseong-gu, Daejeon 34134, Korea; manho1201@nate.com (M.Y.); speed1382@nate.com (G.C.); sksdmlcjf@naver.com (E.H.); j.nam@cnu.ac.kr (J.N.); 2 Research & Development Institute, Lotte Engineering & Construction, 3 Naruteo-ro 10-gil, Seocho-gu, Seoul 06527, Korea; kellery76@gmail.com; 3 Research & Development Institute, Daelim Industrial Co., Ltd., F16 D1, 17 Jongno 3-Gil, Jongno-Gu, Seoul 03155, Korea; ltg777@daelim.co.kr

**Keywords:** high strength concrete, thermal expansion, total strain, transient creep, high temperature, creep strain

## Abstract

Strain is generated in concrete subjected to elevated temperatures owing to the influence of factors such as thermal expansion and design load. Such strains resulting from elevated temperatures and load can significantly influence the stability of a structure during and after a fire. In addition, the lower the water-to-binder (W–B) ratio and the smaller the quantity of aggregates in high-strength concrete, the more likely it is for unstable strain to occur. Hence, in this study, the compressive strength, elastic modulus, and creep behavior were evaluated at target temperatures of 100, 200, 300, 500, and 800 °C for high-strength concretes with W–B ratios of 30%, 26%, and 23%. The loading conditions were set as non-loading and 0.33f_cu_. It was found that as the compressive strength of the concrete increased, the mechanical characteristics deteriorated and transient creep increased. Furthermore, when the point at which creep strain occurred at elevated temperatures after the occurrence of transient creep was considered, greater shrinkage strain occurred as the compressive strength of the concrete increased. At a heating temperature of 800 °C, the 80 and 100 MPa test specimens showed creep failure within a shrinkage strain range similar to the strain at the maximum load.

## 1. Introduction

High-strength concrete (HSC) has a low water-to-binder ratio, which makes its internal structure compact. Therefore, HSC poses a risk of explosive spalling owing to the water vapor pressure that builds up when exposed to elevated temperatures.

Kim, Y.S. and Kim, G.Y. et al. [1], Xiao et al. [2], Liu et al. [3], Peng et al. [4], and Kalifa et al. [5] reported that polypropylene fibers dispersed in concrete melts at 160–170 °C to form micro and macro channels during fires and dissipate the vapor pressure, which enhances the fire resistive performance of structural concrete members.

Yermak et al. [6] and Khaliq et al. [7] reported that steel fibers present within concrete reduce the initiation and progression of micro-cracks and enhance the tensile strength for resisting the vapor pressure triggered by heating, eventually enhancing fire-resistive performance. Furthermore, Pavel et al. [8] and Kim et al. [9] confirmed that the fire-resistant covering material applied to the surface of a concrete member made from aluminosilicates and bottom ash can delay the heat transfer to concrete members during fire, thereby effectively preventing explosive spalling and maintaining strength.

However, even though explosive spalling is prevented by using the various methods as mentioned above, the mechanical characteristics of concrete can still deteriorate owing to the physical and chemical changes in its internal structure, stemming from elevated temperatures.

Yoon M.H. and Kim, G.Y. et al. [10], Xu et al. [11], and Yüzer et al. [12] evaluated the influence of heating temperature on the changes in the mechanical characteristics of concrete. In these studies, the compressive strength or the elastic modulus deteriorated owing to the generation of micro-cracks that are triggered by the thermal decomposition of cement hydrates and the different thermal expansion coefficients of the constituents of the concrete. Such a phenomenon is reportedly prominent at heating temperatures over 500 °C. As described above, the deterioration of mechanical characteristics at elevated temperatures is also an important factor for evaluating concrete at elevated temperature conditions, apart from explosive spalling. Therefore, many researchers evaluated the compressive strength and elastic modulus at elevated temperature under various conditions [13,14,15,16,17].

**Figure 1:**
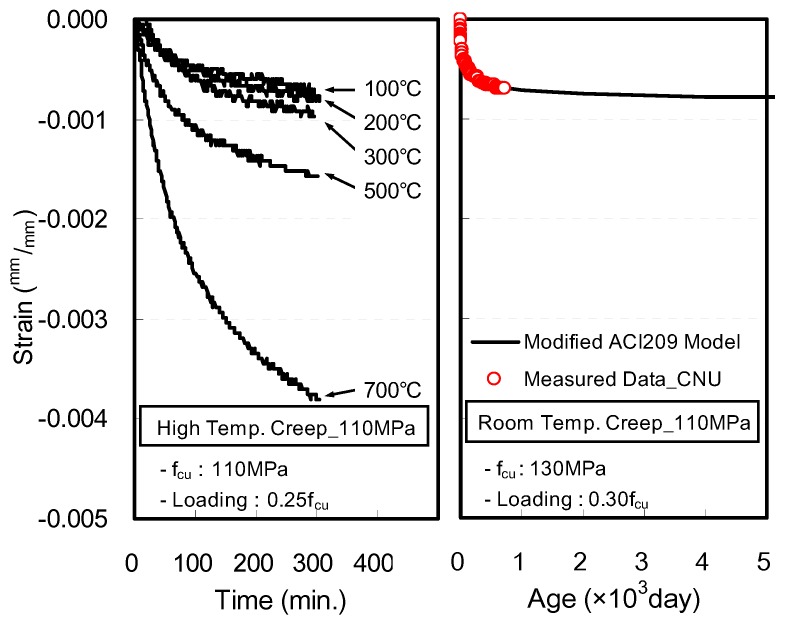
Creep strain of high-strength concrete at ambient and elevated temperatures [18] (f_cu_: compressive strength; CNU: Chungnam National University in Korea; Modified ACI209 Model: ACI209 model modified with CNU data).

Further, strain characteristics such as high-temperature creep, which is generated under constant stress and elevated temperatures, is another important factor to consider for ensuring the stability of a structure after fire, along with explosive spalling and mechanical characteristics at elevated temperatures. Figure 1 [18] shows the creep strains of concrete at room temperature and elevated temperatures. The creep observed in concrete maintained at a temperature of 700 °C for 5 h was 4.5 times greater than that observed in concrete maintained at room temperature for over a year. As such, the concrete specimen under elevated temperature needs to be evaluated for its strain characteristics, which rapidly increase owing to elevated temperatures and loads.

Therefore, in this study, the compressive strength, elastic modulus, thermal strain, total strain, and creep strain of HSC at elevated temperatures were measured to evaluate the influence of the heating temperature and the compressive strength of the concrete on its mechanical characteristics, thermal strain, and creep strain at elevated temperatures, which was not explicitly described in the previous study [18].

## 2. Experimental Plan

### 2.1. Experimental Conditions

Table 1 and Table 2 present the experimental conditions and concrete mix proportions. For HSC specimens with 30%, 26%, and 23% water-to-binder ratio (W–B), the load was set to 33% of the compressive strength at room temperature (0.33f_cu_) to evaluate the compressive strength, elastic modulus, thermal strain, total strain, and transient creep at temperatures of 20, 100, 200, 300, 500, and 800 °C. In addition, under a load of 0.33f_cu_, the specimens were heated up to 200, 400, 600, and 800 °C, while maintaining the given load and temperatures for 5 h to evaluate the creep strain at elevated temperatures. At the time of the heating experiment, the compressive strength of the concrete specimens at room temperature (f_cu_) with W–B ratios of 30%, 26%, the 23% specimens were measured by 74, 80 and 100 MPa, respectively.

Furthermore, to evaluate the characteristics of HSC specimens with 30%, 26%, and 23% W–B at elevated temperatures without the occurrence of explosive spalling, nylon fiber—which was reported to be effective against the explosive spalling of concrete in an existing study—was used to prevent explosive spalling [19].

### 2.2. Materials

The physical properties of the materials used in the experiment are listed in Table 3. Type 1 normal Portland cement, blast furnace slag, and fly ash were used as admixtures. Wash aggregates were used as fine aggregates, while crushed aggregates of granite were used as coarse aggregates.

Furthermore, to prevent the explosive spalling of concrete, nylon fibers with a length of 13 mm, density of 1.10 g/cm^3^, and melting point of 225 °C, were mixed at ratios of 0.045, 0.073, and 0.091 vol%, which are within the mixing range for organic fibers and have a negligible influence on the mechanical performance of concrete at elevated temperatures [5,13].

### 2.3. Specimen Preparation

The ø100 × 200 mm concrete specimens were manufactured in accordance with “ISO 1920-3 Testing of concrete—Part 3: Making and curing test specimens” [20]. The test specimens for measuring compressive strength and elastic modulus at room temperature were cured in 20 ± 2 °C water for 28 days and then evaluated. The heating test specimens were cured under water for 7 days, followed by air dry curing for up to 91 days at a steady temperature and humidity in a chamber set at 20 ± 2 °C, R.H. 50 ± 5%. The upper and lower surfaces of the concrete were smoothly finished with a grinder before the heating test was conducted.

### 2.4. Experimental Method

#### 2.4.1. Heating Apparatus and Method

**Figure 2:**
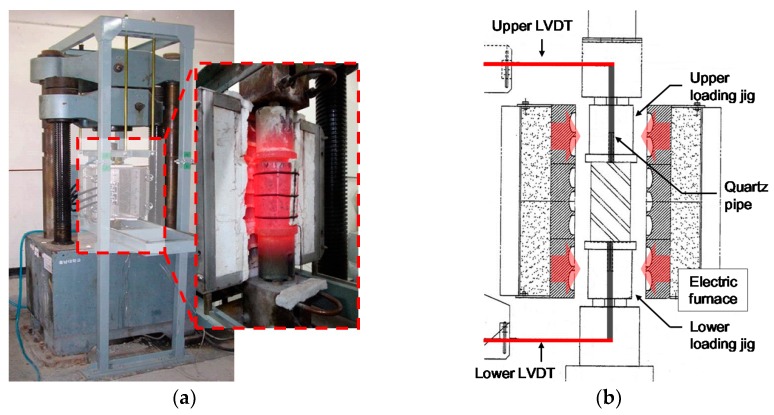
Experimental apparatus: (**a**) heating and loading apparatus; (**b**) geometry of apparatus.

The experimental apparatus used in this study is shown in Figure 2. To simultaneously conduct loading and heating, an electric heating furnace was installed on a 2000-kN-scale loading apparatus. During the measurement of the total strain and creep, the loading and maintenance of the load was controlled by a computer-controlled electrohydraulic servo valve and accuracy ±1% pressure cell.

In addition, the strain of the specimens during heating was measured by delivering the strain to an external displacement meter with a sensitivity of 1000 × 10^−6^ strain/mm via a ø10-mm quartz tube installed at the center of the upper and lower loading jigs. To increase the temperatures on both the inside and outside of the specimen, an indirect heating method, wherein the upper and lower loading jigs were heated, was adopted.

**Figure 3:**
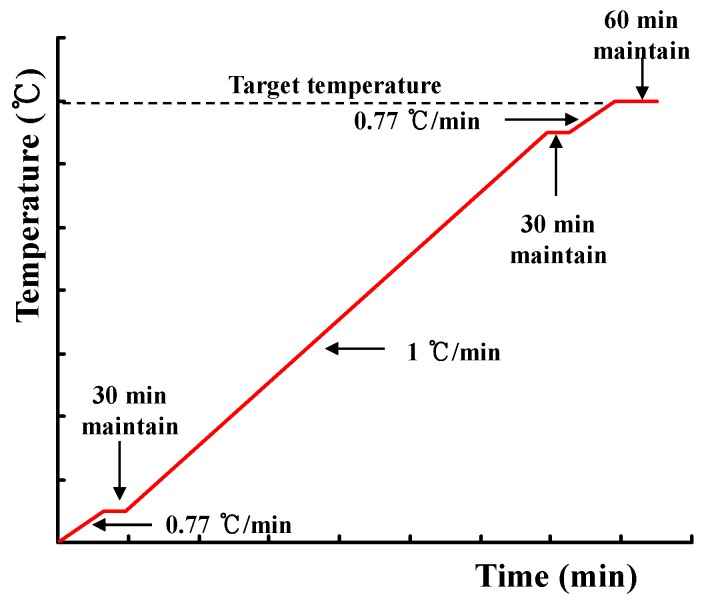
Heating curve used in the experiment.

Further, as shown in Figure 3, the heating was conducted at a rate of 1 °C/min. In particular, based on a previous study [21], the heating rate was set to 0.77 °C/min in the interval from 0 to 50 °C and in the interval from 50 °C before the target temperature to the target temperature. This was done to ensure that the difference between the internal and external temperatures of the specimen was below 5 °C.

#### 2.4.2. Compressive Strength and Elastic Modulus Measuring Method

The compressive strength of concrete at room temperature was measured in accordance with “ASTM C39 Standard Test Method for Compressive Strength of Cylindrical Concrete Specimens” [22]. High temperature compressive strength was measured after heating to the target temperature in the non-loading and 0.33f_cu_ loading condition.

The elastic modulus of the concrete specimen was calculated by the inclination of the point at 40% of the maximum stress and the point of 0.00005 strain in the stress–strain curve according to “ASTM C469 Standard Test Method for Static Modulus of Elasticity and Poisson’s Ratio of Concrete in Compression” [23].

#### 2.4.3. Strain Measuring Method

**Figure 4:**
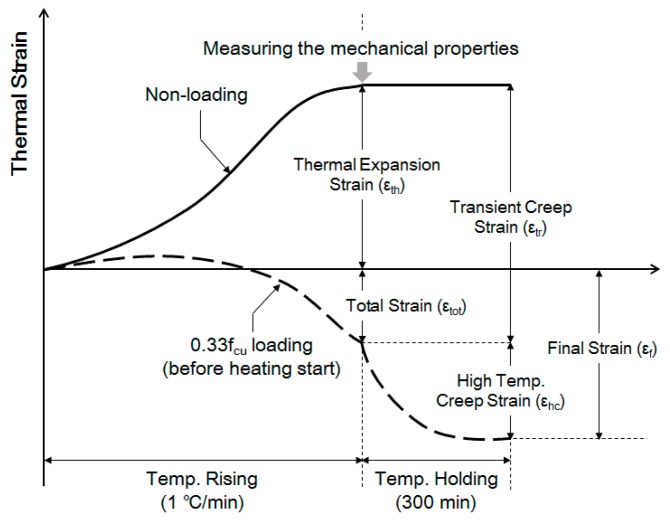
Evaluation of the strain properties of concrete [10].

Figure 4 shows the outline for the strain of concrete during heating and loading. The strain of the specimen was calculated by the length of the specimen measured before and during heating according to the following equation.

(1) $$\varepsilon =\frac{L_{heat}-L_{0}}{L_{0}}$$

where ε denotes the strain, $L_{heat}$ denotes the length of specimen measured during heating, and $L_{0}$ denotes the length of specimen measured before heating.

The strain measured during heating under the non-loading condition was considered as the thermal strain, while the total strain was considered as the strain that occurred during heating under a load of 0.33f_cu_ [24]. The transient creep was calculated as the difference between the thermal strain and total strain.

The creep at elevated temperatures was considered as the strain generated 300 min after reaching the target temperature, under a load of 0.33f_cu_ prior to heating. The final strain was calculated by adding the creep, which was measured when a constant temperature was maintained, to the total strain, which was measured during the time of increasing temperature. The definitions of the items for evaluating strains are expressed as follows:

(2) $$\varepsilon_{tot}=\varepsilon_{th}+\varepsilon_{tr}\;\mathrm{and}$$

(3) $$\varepsilon_{f}=\varepsilon_{tot}+\varepsilon_{hc},$$

where $\varepsilon_{tot}$ denotes the total strain, $\varepsilon_{th}$ denotes the thermal strain, $\varepsilon_{tr}$ denotes the transient creep, $\varepsilon_{f}$. denotes the final strain, and $\varepsilon_{hc}$ denotes the creep strain at elevated temperatures.

## 3. Results and Discussion

### 3.1. Stress–Strain Relation of HSC at Elevated Temperature

**Figure 5:**
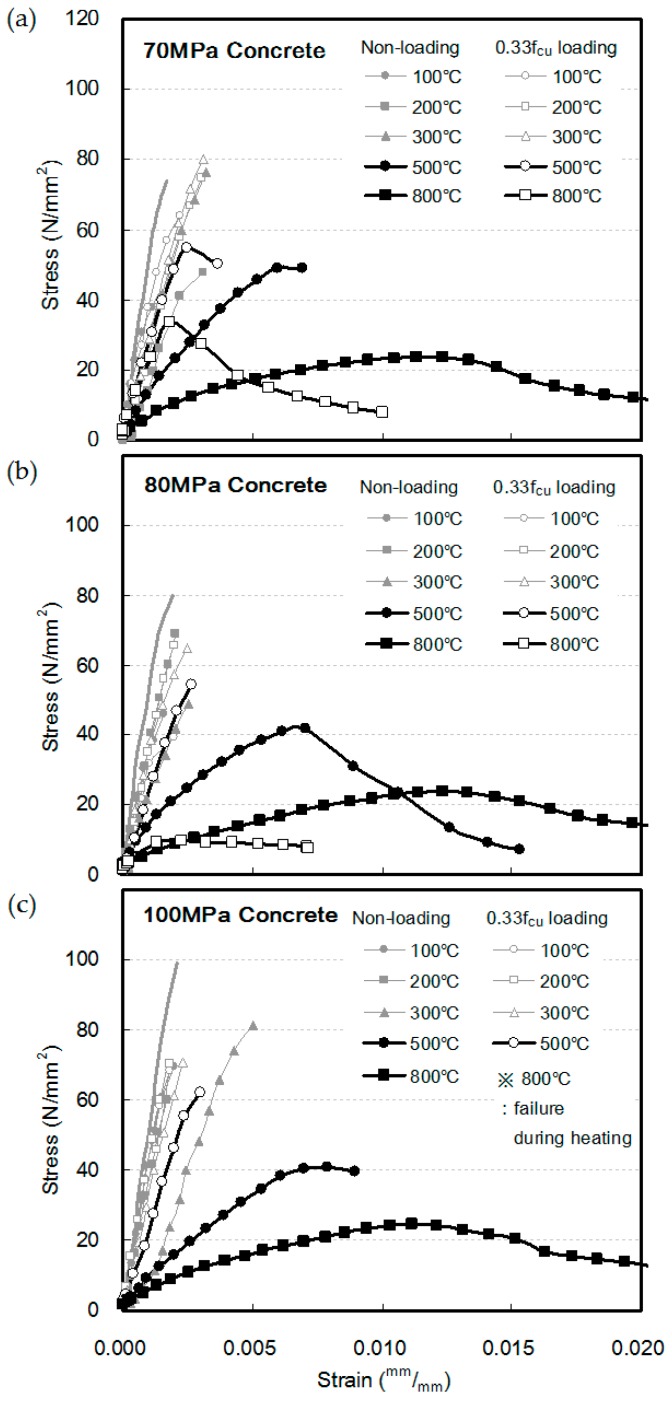
Stress–strain relation of high strength concrete at elevated temperatures: (**a**) 70 MPa; (**b**) 80 MPa; (**c**) 100 MPa.

Figure 5 shows the stress–strain relationship of HSC at elevated temperatures. At heating temperatures 100, 200, and 300 °C, the stress–strain curve showed a linear relationship, regardless of the compressive strength and loading condition of the concrete.

At a heating temperature of 500 °C under the non-loading condition, compared to the case of heating up to 300 °C, the gradient of the stress–strain curve decreased, and the strains of the 70-, 80-, and 100-MPa specimens at the maximum stress were measured as 0.0069, 0.0070, and 0.0079, respectively. In addition, at a heating temperature of 800 °C, all three specimens showed strain at a peak stress above 0.0100, thus significantly decreasing the gradient of the stress–strain curve.

Under a load of 0.33f_cu_, the strains at peak stress for the 70-, 80-, and 100-MPa specimens at 500 °C were 0.0024, 0.0026, and 0.0029, respectively. This confirmed that the strain at peak stress considerably decreases while the maximum stress increases, compared to the non-loading condition. Meanwhile, at 800 °C, the trends in terms of the increasing maximum stress as well as the decreasing strain at the peak stress due caused by loading could not be confirmed. The 70-MPa specimen showed a similar trend to the 500 °C case. However, the 80-MPa specimen showed a maximum stress that was considerably low, and the 100 MPa specimen failed during heating.

### 3.2. Compressive Strength and Elastic Modulus

**Figure 6:**
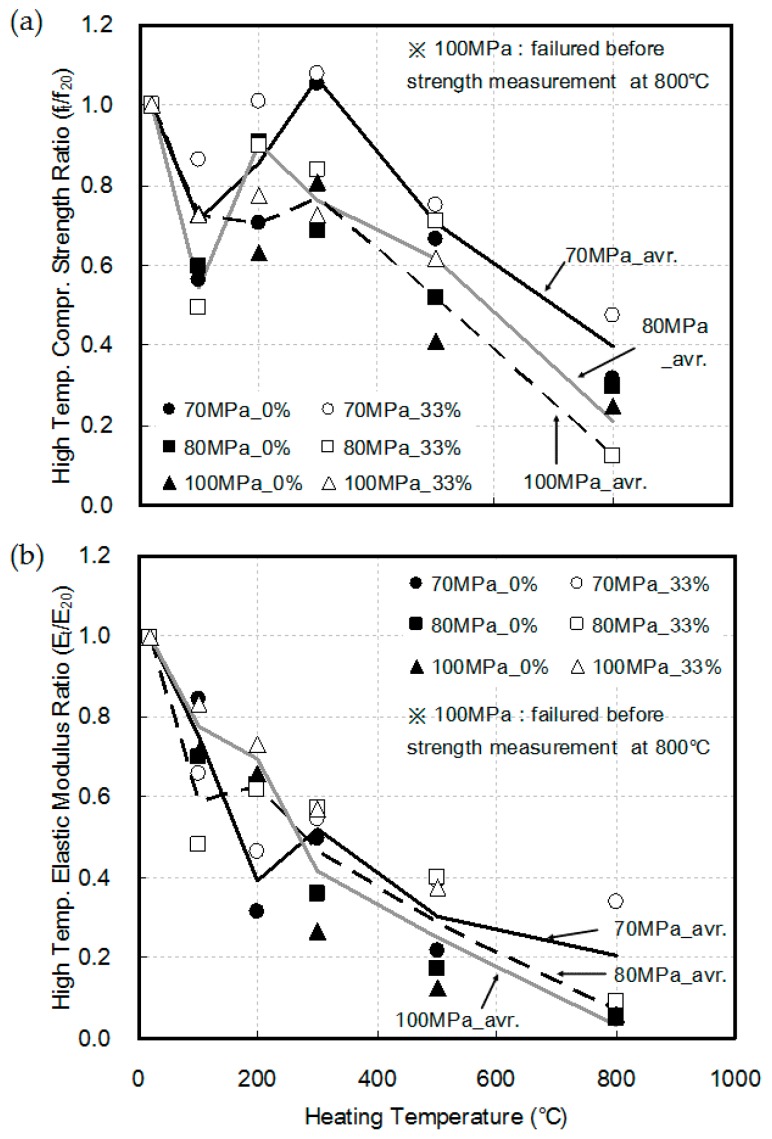
Compressive strength and elastic modulus of high strength concrete at elevated temperatures: (**a**) compressive strength; (**b**) elastic modulus.

Figure 6 shows the compressive strength and elastic modulus of HSC at elevated temperatures. Under a non-loading condition, the compressive strength decreased to approximately 55–63% of the compressive strength at room temperature at a heating temperature of 100 °C, regardless of the W–B ratio of the concrete. This is due to the microcracks generated within the concrete, the evaporation of moisture inside the specimen, and the vapor pressure that followed this, as reported in existing studies [4,10,11,18]. In the heating temperature range of 200–300 °C, the increase in thermal stress by heating and the hydration of unhydrated materials triggered by the hot and pressurized vapor [11] caused the compressive strength to increase again up to 80–110% of the compressive strength at room temperature.

**Figure 7:**
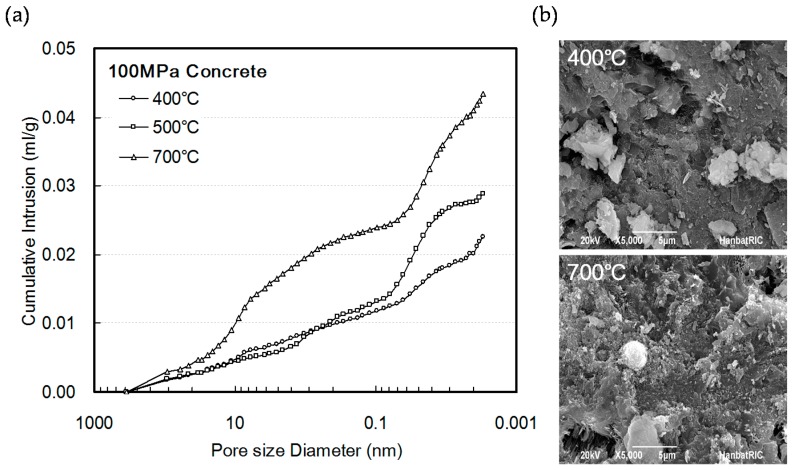
Pore volume and scanning electron microscope image of 100 MPa concrete according to heating temperature: (**a**) cumulative pore volume; (**b**) scanning electron microscope image.

Figure 7a shows the mercury intrusion porosimetry (MIP) measurement results at heating temperatures above 300 °C, thermal decomposition of cement hydrates including the calcium silicate hydrate (CSH) phase, and microcracks generated by the differential thermal expansions of the aggregates and pore expansion caused by cement paste within the concrete. In addition, damage to the cement matrix is also observed in the scanning electron microscope (SEM) measurement results shown in Figure 7b. Therefore, the strength constantly decreased.

Under a load of 0.33f_cu_, the compressive strength at elevated temperatures was greater than that under the non-loading condition, regardless of the W–B ratio of the concrete. This is because the generation and expansion of internal cracks triggered by the expansion of aggregates, known as the major cause of the strength degradation caused by high temperature, are alleviated by the compressive stress caused by the load [25,26].

**Figure 8:**
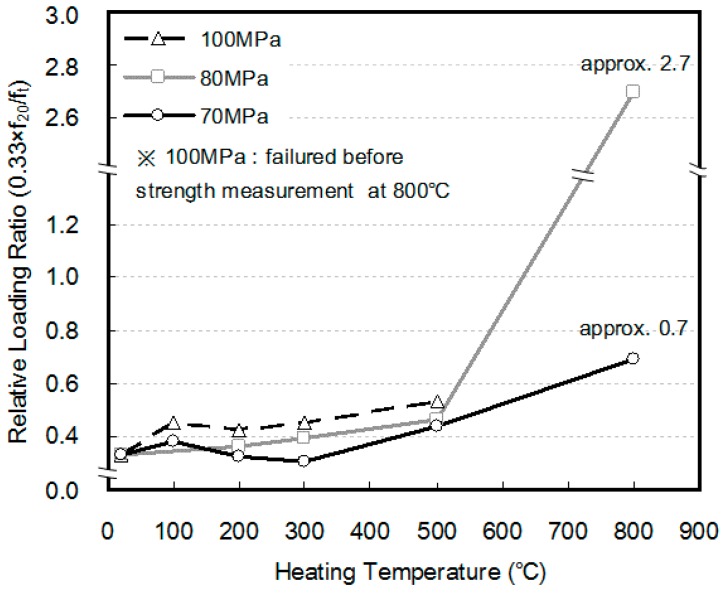
Relative loading ratio of HSC.

However, at a heating temperature of 800 °C, the 80 MPa specimen showed lower compressive strength than that under the non-loading condition, while the 100 MPa specimen failed before its compressive strength could be measured. To further explain the cause of failure, the amount of relative loading changes with the changes in the heating temperature as well as the compressive strength were compared, as shown in Figure 8. Although the load was 0.33f_cu_ prior to heating, the relative ratio of loading increased owing to the decrease in compressive strength caused by the increasing temperature. Furthermore, the higher the strength of the concrete, the more the compressive strength was likely to decrease. Therefore, the rate of relative loading was confirmed to be higher. At a heating temperature of 800 °C, the 80 MPa specimen showed lower compressive strength than that observed under the non-loading condition, while the 100 MPa specimen failed before its compressive strength could be measured because of the loading weight.

As shown in Figure 6b, the value of the elastic modulus constantly decreased as the heating temperature increased. Similar to compressive strength at elevated temperatures, the value of the elastic modulus under the loading condition was higher, than that under the non-loading condition. Further, the higher the strength of the concrete, the ratio of the cement matrix that had a large influence on the strength was higher than that of the aggregate. Therefore, by thermal decomposition of the cement matrix, the more likely it is for the elastic modulus to decrease with increasing temperature, although the difference was not significant.

### 3.3. Thermal Expansion and Total Strain

**Figure 9:**
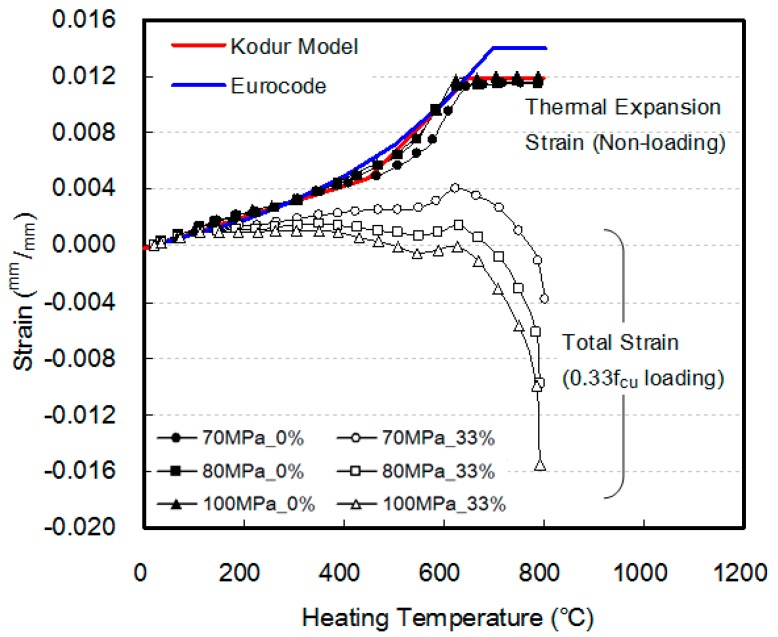
Thermal expansion and total strain of HSC.

Figure 9 shows the thermal expansion strain and the total strain with respect to the heating temperature. The thermal expansion strain of the HSC as measured under the non-loading condition, at heating temperatures below 600 °C, exhibited values similar to those of Eurocode [27] and Kodur’s model [14], irrespective of the compressive strength of the concrete. However, at heating temperatures above 600 °C, the results of this study and Kodur’s model converged to 0.012, while the results from Eurocode converged to 0.014 at 700 °C. Therefore, at heating temperatures above 600 °C, Eurocode modeled the thermal expansion deformation more or less excessively.

Under a load of 0.33f_cu_, the total exhibited showed abrupt shrinkage at a heating temperature of 600 °C. Furthermore, the shrinkage strain increased as the compressive strength of the concrete increased. This is because the relative ratio of the loaded weight increases as the deterioration in compressive strength increases, owing to the elevated temperature when the compressive strength of concrete increases, as explained in Figure 7.

**Figure 10:**
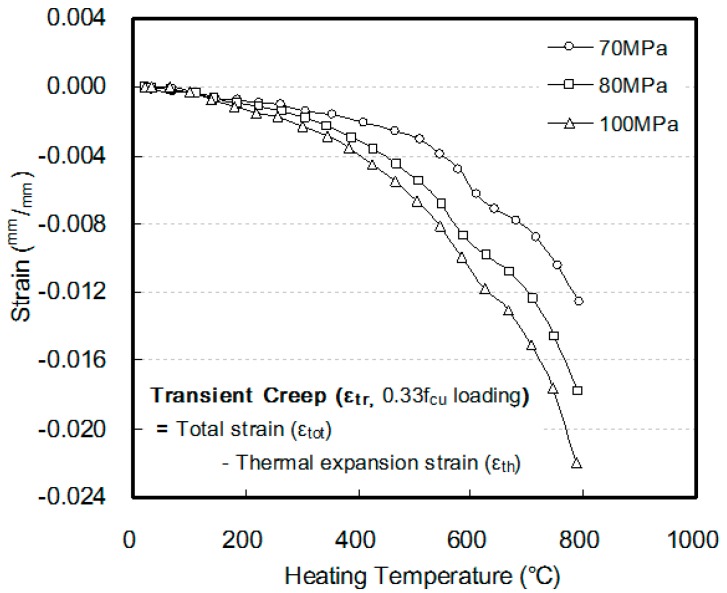
Transient creep of HSC.

Figure 10 shows the transient creep according to the compressive strength of concrete and the heating temperature. Sadaoui et al. [28] reported that transient creep occurs owing to thermo-mechanical interaction in concrete exposed to elevated temperatures, which influences the rapid strain behavior of concrete at elevated temperatures. In this study, in all the specimens, the shrinkage strain increased as the heating temperature increased, and the increment was rapid within the temperature range of 500–600 °C. This is caused by the internal cracks that are generated owing to rapid expansion, which is triggered by the phase variation (at approximately 570 °C) of quartz included within the aggregates [29,30]. In addition, because the relative ratio of load increased, the amount of shrinkage increased as the compressive strength of concrete further increased.

### 3.4. High Temperature Creep and Final Strain

**Figure 11:**
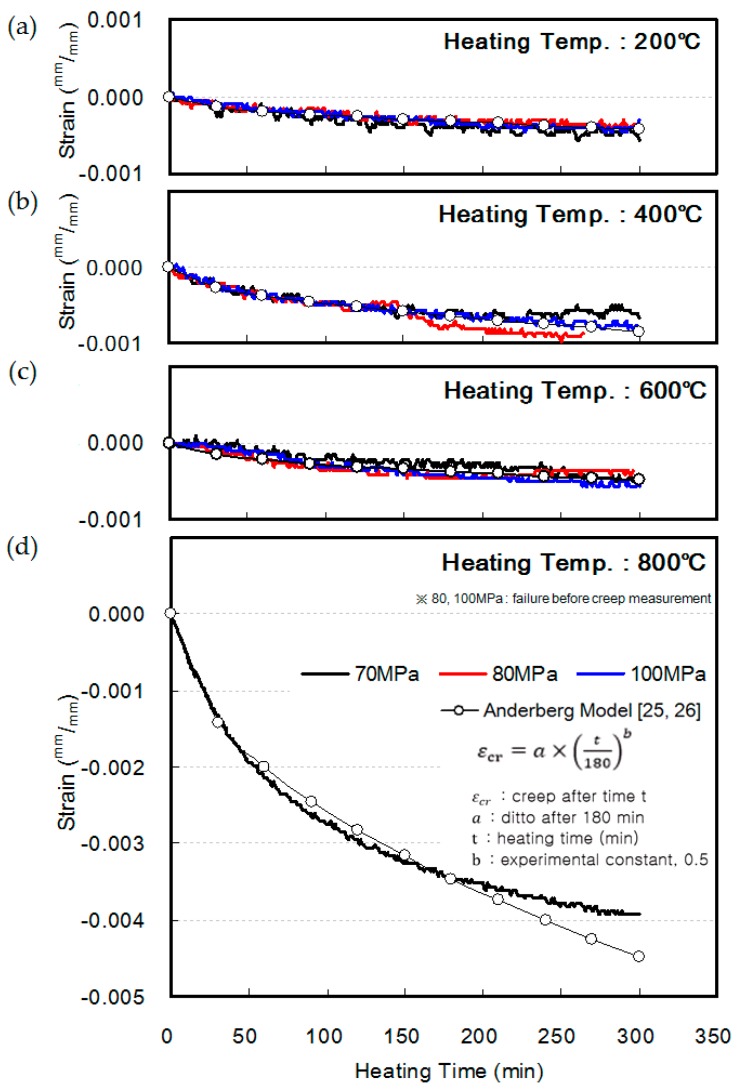
Creep of HSC at elevated temperature: (**a**) 200 °C; (**b**) 400 °C; (**c**) 600 °C; (**d**) 800 °C.

Figure 11 shows the creep behavior of HSC at elevated temperatures, measured while maintaining the load and heating temperature. At heating temperatures of 200, 400, and 600 °C, the respective creeps were −0.0040, −0.007 to −0.010, and −0.0045, showing no significant difference according to the compressive strength of concrete. In addition, the results were similar to those obtained using the Anderberg model proposed in previous studies [31,32].

Meanwhile, at a heating temperature of 800 °C, the 70 MPa specimen showed a significant increase in shrinkage strain, reaching a value of approximately −0.004, 300 min after the measurement. However, the 80 MPa and 100 MPa specimens reached failure before their creep could be measured.

**Figure 12:**
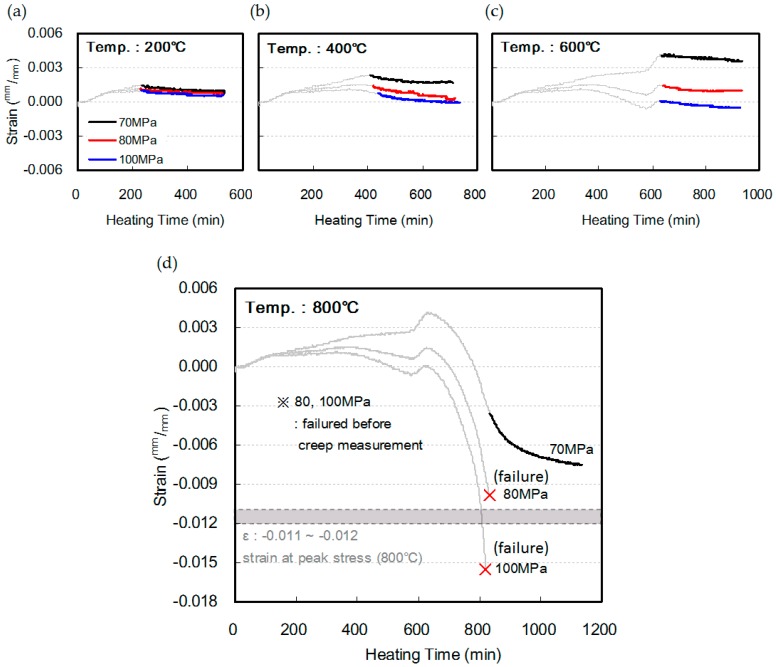
Final strain of HSC at elevated temperature: (**a**) 200 °C; (**b**) 400 °C; (**c**) 600 °C; (**d**) 800 °C.

Unlike the trend in which the amount of shrinkage relatively increased at elevated temperatures along with an increase in the compressive strength of concrete, the creep was similar regardless of the compressive strength of the concrete within the heating temperature range of 200–600 °C. This can be explained as the final strain, which reflects the creep after the total strain. As shown in Figure 12, a greater shrinkage strain was observed as the compressive strength of concrete increased. In particular, at a heating temperature of 600 °C, the shrinkage strains of the 70, 80, and 100 MPa specimens were 0.0036, 0.0010, and −0.0005, respectively, thereby clearly confirming the increasing trend of shrinkage strain with the increase in the compressive strength of concrete. Therefore, although the creep was similar regardless of the compressive strength, because the transient creep becomes larger as the compressive strength of concrete increases, a larger shrinkage strain was observed.

At a heating temperature of 800 °C—except for the 70 MPa specimen, which showed a strain of −0.0075—the 80 and 100 MPa specimens reached failure at strains of −0.0098 and −0.0155, respectively, which are similar to or slightly higher than −0.011 to −0.012, which were the strains measured at the maximum load during the measurement of compressive strength. Therefore, it was found that a greater shrinkage strain occurs owing to heating and loading as the compressive strength of concrete increases, and that failure can occur owing to creep, once this shrinkage strain reaches the fracture strain of concrete.

In some existing studies [31,32], the creep of concrete at elevated temperatures is simply expressed as the creep value according to time. However, as shown by the results of this study, in order to evaluate the strain behavior of concrete at elevated temperatures, in addition to the creep at elevated temperatures, the transient creep at the interval of rising temperatures should be considered for the evaluation of the final strain.

## 4. Conclusions

1. In this study, transient creep, which is generated upon heating an HSC specimen loaded with a predetermined amount of load (0.33f_cu_), and creep at elevated temperatures, which is generated upon maintaining a certain level of temperature for a certain amount of time, were measured in order to understand the strain behavior that occurs in HSC at loaded and heated conditions.
2. Within the scope of this study, the amount of creep of HSC at elevated temperatures showed similar results at respective heating temperature levels, regardless of the compressive strength. However, when the point at which creep occurs at elevated temperatures after the occurrence of transient creep was considered, a larger shrinkage strain was observed as the compressive strength of concrete increased.
3. At a heating temperature of 800 °C, the 80 and 100 MPa specimens reached failure when their final strains were similar to or slightly higher than the strain at the maximum load during the measurement of compressive strength at 800 °C. Therefore, it was found that as the compressive strength of concrete increases, an even larger shrinkage strain occurs owing to heating and loading, which in turn can lead to creep failure during heating.
4. In order to guarantee the strain stability of a structure made of HSC during and after a fire, the destruct limit state, thermal expansion in all heating segments, total strain, transient creep, and creep of concrete at elevated temperature should be considered.

## Figures and Tables

**Table 1:** Experimental conditions.

ID (f_ck_^1^)	Fiber Mixing Ratio (vol.%)	Pre-Loading Level (×f_cu_)	Target Temp. (°C) ^2^	Heating VeloCity (°C/min)	Properties Evaluated
70 MPa	0.045	0.00 0.33	20, 100, 200, 300, 500, 800	1	compressive strength and elastic modulus thermal expansion and total strain creep strain
80 MPa	0.073				
100 MPa	0.091				

^1^ f_ck_: design compressive strength of concrete, ^2^ target temperature of creep strain measurement: 200, 400, 600, and 800 °C.

**Table 2:** Concrete mix proportion.

ID (f_ck_)	W–B ^1^ (%)	Slump-Flow (mm)	Air (%)	S/a ^2^ (%)	Unit Weight (kg/m^3^) ^3^							
					W	C	GGBS	FA	S	G	Fiber	SP
70 MPa	30	650 ± 50	2 ± 1	49	164	380	136	27	810	860	0.6	3.9
80 MPa	26			47	163	392	180	50	743	854	0.8	4.5
100 MPa	23			46		399	210	91	718	859	1.0	8.4

^1^ W–B: water-to-binder ratio, ^2^ S/a: the ratio of fine aggregate volume to the total aggregate volume, ^3^ W: water; C: cement; GGBS: ground granulated blast furnace slag; FA: fly ash; S: fine aggregate; G: coarse aggregate; Fiber: Nylon fiber; SP: super plasticizer.

**Table 3:** Physical properties of the materials.

Materials	Physical Properties
Cement	Ordinary Portland Cement Density: 3.15 g/cm^3^, Specific surface area: 3630 cm^2^/g
Fine aggregate	Washed sand Density: 2.60 g/cm^3^, Water absorption ratio: 1.03%
Coarse aggregate	Crushed granite Max size: 20 mm, Density: 2.62 g/cm^3^, Water absorption ratio: 0.97%
Fly ash	Density: 2.20 g/cm^3^, Specific surface area: 4600 cm^2^/g
Ground granulated blast furnace slag	Density: 2.90 g/cm^3^, Specific surface area: 4530 cm^2^/g
Nylon fiber	Density: 1.10 g/cm^3^, Length: 13 mm, Melting point: 225 °C
Admixture	Polycarboxylic water reducing agent

## References

[1] Kim Y.-S., Lee T.-G., Kim G.-Y. (2012). An experimental study on the residual mechanical properties of fiber reinforced concrete with high temperature and load. Mater. Struct..

[2] Xiao J., Falkner H. (2006). On residual strength of high-performance concrete with and without polypropylene fibres at elevated temperatures. Fire Saf. J..

[3] Liu X., Ye G., De Schutter G., Yuan Y., Taerwe L. (2008). On the mechanism of polypropylene fibres in preventing fire spalling in self-compacting and high-performance cement paste. Cem. Concr. Res..

[4] Peng G.-F., Yang W.-W., Zhao J., Liu Y.-F., Bian S.-H., Zhao L.-H. (2006). Explosive spalling and residual mechanical properties of fiber-toughened high-performance concrete subjected to high temperatures. Cem. Concr. Res..

[5] Kalifa P., Chéné G., Gallé C. (2001). High-temperature behaviour of hpc with polypropylene fibres: From spalling to microstructure. Cem. Concr. Res..

[6] Yermak N., Pliya P., Beaucour A.L., Simon A., Noumowé A. (2017). Influence of steel and/or polypropylene fibres on the behaviour of concrete at high temperature: Spalling, transfer and mechanical properties. Constr. Build. Mater..

[7] Khaliq W., Kodur V. (2011). Thermal and mechanical properties of fiber reinforced high performance self-consolidating concrete at elevated temperatures. Cem. Concr. Res..

[8] Krivenko P.V., Guzii S.G., Bodnarova L., Valek J., Hela R., Zach J. (2016). Effect of thickness of the intumescent alkali aluminosilicate coating on temperature distribution in reinforced concrete. J. Build. Eng..

[9] Kim J.-H.J., Lim Y.M., Won J.P., Park H.G. (2010). Fire resistant behavior of newly developed bottom-ash-based cementitious coating applied concrete tunnel lining under rabt fire loading. Constr. Build. Mater..

[10] Yoon M., Kim G., Choe G.C., Lee Y., Lee T. (2015). Effect of coarse aggregate type and loading level on the high temperature properties of concrete. Constr. Build. Mater..

[11] Xu Y., Wong Y.L., Poon C.S., Anson M. (2001). Impact of high temperature on PFA concrete. Cem. Concr. Res..

[12] Yüzer N., Aköz F., Öztürk L.D. (2004). Compressive strength–color change relation in mortars at high temperature. Cem. Concr. Res..

[13] Poon C.S., Shui Z.H., Lam L. (2004). Compressive behavior of fiber reinforced high-performance concrete subjected to elevated temperatures. Cem. Concr. Res..

[14] Kodur V.K.R., Sultan M.A. (2003). Effect of temperature on thermal properties of high-strength concrete. J. Mater. Civ. Eng..

[15] Abdulkareem O., Abdullah M., Hussin K., Ismail K., Binhussain M. (2013). Mechanical and microstructural evaluations of lightweight aggregate geopolymer concrete before and after exposed to elevated temperatures. Materials.

[16] Heap M.J., Lavallée Y., Laumann A., Hess K.U., Meredith P.G., Dingwell D.B., Huismann S., Weise F. (2013). The influence of thermal-stressing (up to 1000 °C) on the physical, mechanical, and chemical properties of siliceous-aggregate, high-strength concrete. Constr. Build. Mater..

[17] Dong H., Cao W., Bian J., Zhang J. (2014). The fire resistance performance of recycled aggregate concrete columns with different concrete compressive strengths. Materials.

[18] Lee Y.W., Kim G.Y., Gucunski N., Choe G.C., Yoon M.H. (2015). Thermal strain behavior and strength degradation of ultra-high-strength-concrete. Mater. Struct..

[19] Lee G., Han D., Han M.-C., Han C.-G., Son H.-J. (2012). Combining polypropylene and nylon fibers to optimize fiber addition for spalling protection of high-strength concrete. Constr. Build. Mater..

[20] International Organization for Standardization (2004). Testing of Concrete—Part 3: Making and Curing Test Specimens.

[21] Kim G.-Y., Kim Y.-S., Lee T.-G. (2009). Mechanical properties of high-strength concrete subjected to high temperature by stressed test. Trans. Nonferrous Met. Soc. China.

[22] American Society for Testing Materials (2015). Standard Test Method for Compressive Strength of Cylindrical Concrete Specimens.

[23] American Society for Testing Materials (2014). Standard Test Method for Static Modulus of Elasticity and Poisson's Ratio of Concrete in Compression.

[24] RILEM TC 129-MHT (2000). Test methods for mechanical properties of concrete at high temperatures Part 8 Steady-state creep and creep recovery for service and accident conditions. Mater. Struct..

[25] Arioz O. (2007). Effects of elevated temperatures on properties of concrete. Fire Saf. J..

[26] Zega C.J., Di Maio A.A. (2009). Recycled concrete made with different natural coarse aggregates exposed to high temperature. Constr. Build. Mater..

[27] British Standards Institution (2005). Eurocode 2: Design of Concrete Structures. General Rules. Structural Fire Design.

[28] Sadaoui A., Khennane A. (2009). Effect of transient creep on the behaviour of reinforced concrete columns in fire. Eng. Struct..

[29] Niry Razafinjato R., Beaucour A.-L., Hebert R.L., Ledesert B., Bodet R., Noumowe A. (2016). High temperature behaviour of a wide petrographic range of siliceous and calcareous aggregates for concretes. Constr. Build. Mater..

[30] Savva A., Manita P., Sideris K.K. (2005). Influence of elevated temperatures on the mechanical properties of blended cement concretes prepared with limestone and siliceous aggregates. Cem. Concr. Compos..

[31] Anderberg Y., Thelandersson S. (1976). Stress and Deformation Characteristics of Concrete at High Temperatures. 2. Experimental Investigation and Material Behaviour Model.

[32] Li L.-Y., Purkiss J. (2005). Stress–strain constitutive equations of concrete material at elevated temperatures. Fire Saf. J..

